# Hypomagnesemia in intestinal lymphangiectasia: a case report and review of the literature

**DOI:** 10.1186/s12876-022-02318-6

**Published:** 2022-05-15

**Authors:** Hao Feng, Linfeng Zou, Xiao Zhai, Shengyu Zhang, Jingnan Li

**Affiliations:** 1 Department of Dermatology, State Key Laboratory of Complex Severe and Rare Diseases, Peking Union Medical College Hospital, Chinese Academy of Medical Sciences, Peking Union Medical College, National Clinical Research Center for Dermatologic and Immunologic Diseases, Beijing, China; 2 Department of Gastroenterology, State Key Laboratory of Complex Severe and Rare Diseases, Peking Union Medical College Hospital, Chinese Academy of Medical Sciences and Peking Union Medical College, No. 1 Shuaifuyuan, Dongcheng District, Beijing, 100730 China; 3 Department of Endocrinology, State Key Laboratory of Complex Severe and Rare Diseases, Peking Union Medical College Hospital, Chinese Academy of Medical Sciences, Peking Union Medical College, Beijing, China

**Keywords:** Intestinal lymphangiectasia, Waldmann’s disease, Protein-losing enteropathy, Hypoproteinemia, Case report

## Abstract

**Background:**

Intestinal lymphangiectasia (IL) is a rare disease characterized by dilation of lymphatic vessels and leakage of lymphatic fluids into the intestinal lumen, causing depletion of lymphocytes, protein, lipids, fat-soluble vitamins, and electrolytes. Hypomagnesemia can occur in IL patients but is seldom discussed.

**Case presentation:**

A 30-year-old Tibetan woman who had chronic diarrhea, edema, tetany, and tingling was diagnosed with IL. Prominent hypomagnesemia was noticed. She was treated with a medium-chain triglyceride (MCT) diet and nutrient supplementation with satisfactory results. We also present a systematic review of hypomagnesemia in IL cases from the published literature.

**Conclusions:**

Hypomagnesemia may be an overlooked complication of IL, thus monitoring serum magnesium concentrations in IL patients is crucial.

## Background

Intestinal lymphangiectasia (IL) is a rare disease with unknown prevalence and is characterized by dilated lymphatic vessels resulting in the leakage of lymphatic fluids into the intestinal lumen [1]. Primary IL has an early onset with undetermined etiology, whereas secondary IL is associated with preexisting conditions, including myxedema heart, systemic lupus erythematosus, radiotherapy/chemotherapy, Waldenstrom’s macroglobulinemia, primary peritoneal carcinoma, diffuse large B-cell lymphoma, and primary hypoparathyroidism [2]. Clinical presentations often include diarrhea/vomiting, edema, third cavity fluid accumulation, and recurrent infections. While hypoalbuminemia, hypogammaglobulinemia, lymphopenia, and hypocalcemia are well-known laboratory findings for IL, little is known about the magnesium levels in IL patients [1]. Absorption of magnesium in the small intestine plays an important part in its homeostasis, along with skeletal storage and renal excretion. In addition, one-third of the active form (as opposed to the storage form) of body magnesium is bound to albumin [3]. Thus, disturbed absorption and lowered albumin levels in IL patients may put these patients at a risk of magnesium depletion. Here, we present an intestinal lymphangiectasia case in which prominent hypomagnesemia was present and perform a systematic review of the published literature on this issue.

## Case presentation

### Anamnesis

A 30-year-old Tibetan woman was admitted for recurrent diarrhea, edema, tetany, and tingling for more than 10 years. Her symptoms first appeared when she was a teenager, and fatty meals caused diarrhea, edema of her face and extremities, and tingling. The symptoms were undiagnosed and lasted for two years before disappearing spontaneously. Three years ago, approximately 1 month after giving birth to her first baby, the same symptoms recurred. At first, her diarrhea was triggered by fatty meals, but gradually, she began to suffer from watery stool 3–6 times a day despite a fat-restricted diet. With worsening diarrhea, the patient gradually developed continuous facial and peripheral edema, a constant tingling sensation all over her body and tetany. Her past medical history revealed hepatitis B, total nephrectomy of her left kidney and subsequent chemotherapy and radiotherapy at the age of three due to a “kidney tumor”.

### Clinical findings

The patient had a body mass index (BMI) of 19.5 kg/m^2^. On physical examination, she was otherwise normal except for a prominent forehead, peripheral pitting edema and abdominal shifting dullness. Laboratory examination revealed lymphopenia, hypoalbuminemia, hypogammaglobulinemia, hypokalemia, hypocalcemia, hypophosphatemia, and hypomagnesemia (Table 1). The high-sensitivity C-reactive protein (hsCRP) level and erythrocyte sedimentation rate (ESR) were within normal limits. Screening of fat-soluble vitamins showed decreased serum vitamin A, vitamin D and vitamin E at the lower limit. Routine urine and stool analyses were normal. Electrocardiogram (ECG) showed a prolonged QT interval at 445 ms.

**Table 1:** Laboratory findings for the patient

	Laboratory tests	Results	Normal range
CBCs	WBC (×10^9^/L)	3.03	3.50–9.50
	LY (×10^9^/L)	0.19	0.80-4.00
	LY%	6.3%	20.0–40.0
	HGB (g/L)	111	110–150
	PLT (×10^9^/L)	232	100–350
LFTs	ALT (IU/L)	33	7–40
	AST (IU/L)	29	13–35
	Total bilirubin (mmol/L)	5	5.1–22.2
	Direct bilirubin (mmol/L)	1.7	0.0-6.8
	PT (s)	12.9	10.4–12.6
RFTs	Creatinine (µmol)	38	45–84
	Urea (mmol/L)	5.13	2.78–7.14
Electrolytes	Sodium (mmol/L)	141	135–145
	Potassium (mmol/L)	3.1	3.50–5.50
	Calcium (mmol/L)	1.35	2.13–2.70
	Corrected calcium (mmol/L)	1.77	2.13–2.70
	Phosphorus (mmol/L)	0.23	0.81–1.45
	Iron (µg/dl)	71	50–170
	Magnesium (mmol/L)	0.37	0.70–1.10
Biochemistry	Total protein (g/L)	26	60–85
	Albumin (g/L)	19	35–52
	Vitamin A (mg/L)	0.22	0.33–0.78
	Total 25-(OH)-Vitamin D (ng/ml)	8.2	30–100
	Vitamin E (mg/L)	5.8	5.5–17.0
Immunology	IgG (g/L)	2.73	7.00–17.00
	IgA (g/L)	0.47	0.70-4.00
	IgM (g/L)	0.29	0.40–2.30
	ANA	Negative	Negative
Lipids	Cholesterol (mmol/L)	3.65	2.85–5.70
	LDL (mmol/L)	2.59	< 3.37
	HDL (mmol/L)	0.65	0.93–1.81
	TG (mmol/L)	0.94	0.45–1.70

*CBC* cell blood count, *WBC* white blood cell, *HGB* hemoglobin, *PLT* platelet, *RFT* renal function test, *LFT* liver function test, *ALT* alanine aminotransferase, *AST* aspartate aminotransferase, *PT* prothrombin time, *ANA* antinuclear antibody, *IgG* immunoglobulin G, *IgA* immunoglobulin A; *IgM* immunoglobulin M, *LDL* low-density lipoprotein, *HDL* high-density lipoprotein, *TG* triglycerides

### Diagnostic focus and assessment

**Fig. 1:**
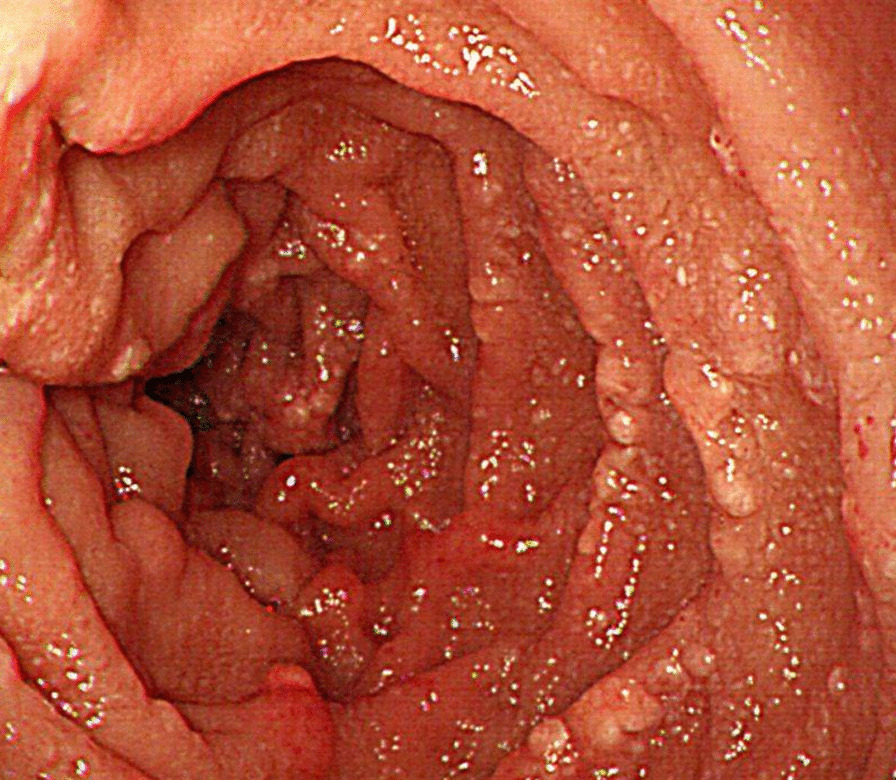
Whitish granules in the 2nd part of the duodenum seen by endoscopy

The marked hypoalbuminemia account for her edema, and the hypocalcemia account for her tetany and tingling. The diarrhea seemed to be the culprit of the hypoalbuminemia since there was no evidence of liver synthesis dysfunction or renal protein leakage. Protein-losing gastroenteropathy was suspected, and the common etiologies of protein-losing gastroenteropathy may include inflammatory bowel disease, infection and IL. While normal hsCRP, ESR and stool analysis made the former two differential diagnoses unlikely, lymphatic imaging using a ^99m^ technetium-labeled tracer showed lymph leakage into the intestines. Endoscopically, there were whitish granules in the descending duodenum and the terminal ileum (Fig. 1), which were later proven to be dilated lymphatic vessels by pathology evaluation. IL secondary to heart dysfunction, portal vein hypertension or malignancy obstruction was ruled out by normal findings on echocardiogram and thoracic/abdominal/pelvic computed tomography scanning, respectively. The patient was eventually diagnosed with IL, which was probably secondary to radiotherapy/chemotherapy of the retroperitoneal region. Her IL was complicated by nutrient deficiencies, including hypomagnesemia.

### Therapeutic focus and outcome

**Fig. 2:**
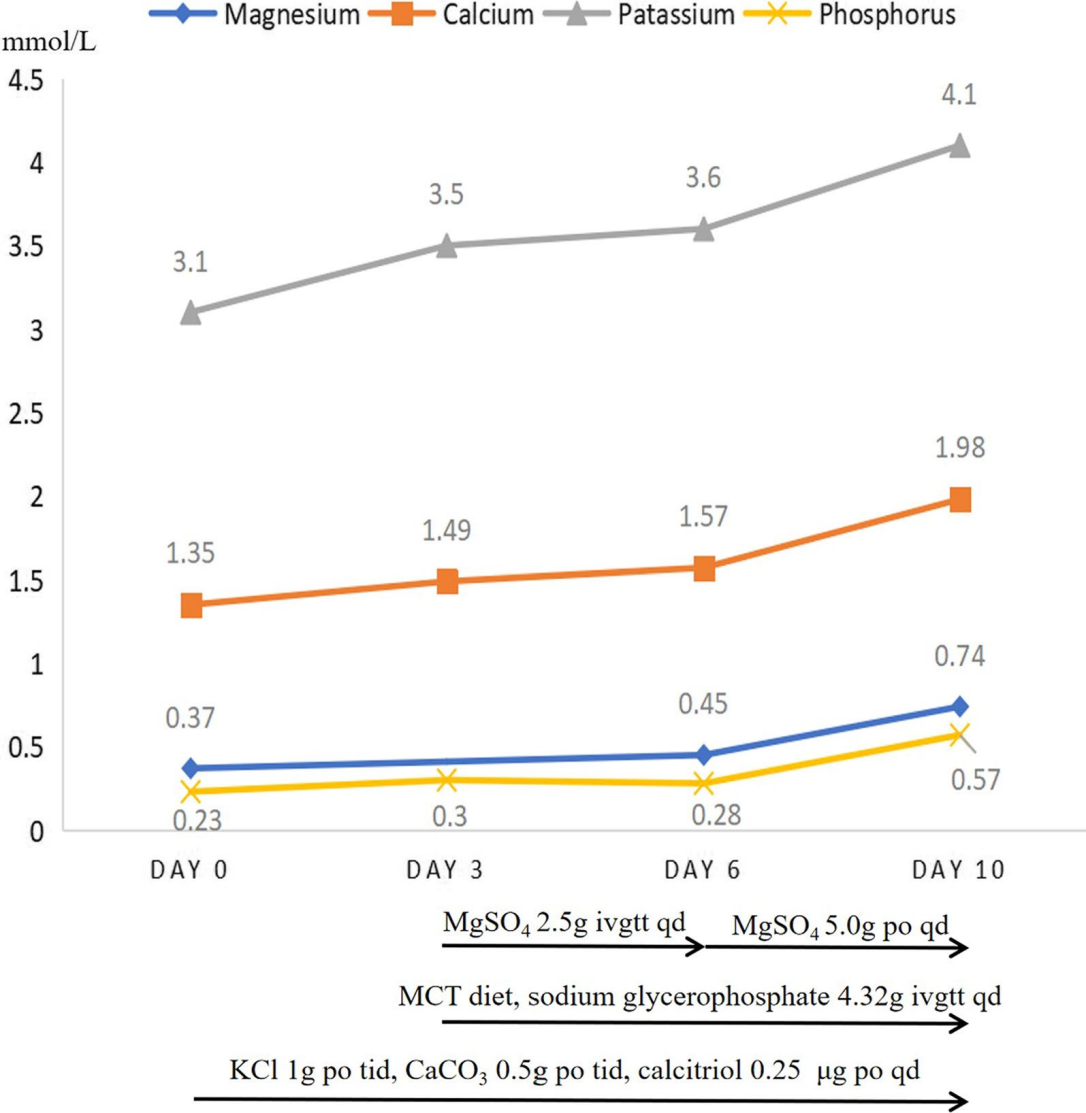
Treatment and changes in electrolytes. A medium chain triglyceride diet and mineral supplements were given to restore the electrolyte balance. Potassium chloride, calcium carbonate, and calcitriol were given from Day 0, and a medium chain triglyceride diet, intravenous magnesium sulfate, and intravenous sodium glycerophosphate were given from Day 3. From Day 6, magnesium sulfate was given orally. The electrolyte status gradually improved. KCl, potassium chloride; CaCO_3_, calcium carbonate; MgSO_4_, magnesium sulfate; MCT, medium chain triglyceride; g, gram; IV, intravenous drip; po, orally; qd, once daily; tid, three times a day

A strict MCT diet was given, and her diarrhea and edema gradually improved, with serum albumin rising to 25 g/L. Oral calcium, calcitriol, potassium supplements were given, as well as intravenous sodium glycerophosphate, which restored serum calcium to 1.98 mmol/L (2.28 after correction for albumin level), potassium to 4.1 mmol/L, and phosphorus to 0.57 mmol/L (Fig. 2). The tetany and tingling all disappeared. For her hypomagnesemia, intravenous magnesium sulfate was given for 3 days before the serum magnesium level rose to 0.45 mmol/L, followed by oral magnesium sulfate for another 3 days, which brought her magnesium level to 0.74 mmol/L before discharge.

## Discussion and conclusion

Depletion of multiple materials in the blood is expected with excessive loss of the nutrient-rich lymph in IL patients. Lymphopenia, hypoalbuminemia, and hypogammaglobulinemia are common laboratory findings in IL. Iron, calcium, lipids, and fat-soluble vitamins may also be depleted. We believe this case of hypomagnesemia in a patient with IL is unique because few IL reports provide information on serum magnesium levels, and even fewer address the treatment of hypomagnesemia.

The prominent hypomagnesemia in our case alerted us to investigate the literature on this issue. Out of the 274 PubMed database retrievable IL cases where biochemical laboratory results were provided, only 23 cases (8%) reported the serum magnesium concentration (Table 2) [2, 4–23].

**Table 2:** Summary of published IL cases with serum magnesium concentration results

Authors	Age/sex	Clinical manifestations	Magnesium (mmol/L)	Total calcium (mmol/L)	Corrected calcium* (mmol/L)	Albumin (g/L)	Potassium (mmol/L)	Primary/secondary to	Managements
Alshikho et al. [2]	24 years/M	Diarrhea, abdominal pain, nausea/vomiting, edema, recurrent infection	0.85	1.98	2.36	21	3.8	Primary	MCT diet, octreotide
Altın et al. [4]	34 years/F	Dyspnea, edema	↓	–	–	↓	↓	Primary	MCT diet, octreotide
Lu et al. [5]	34 years/F	Diarrhea, nausea/vomiting, edema, tetany	0.53	1.72	2.13	19.6	–	Primary	MCT diet, IV albumin
O’Donnell et al. [6]	16 years/F	Diarrhea, edema, peripheral paresthesia, seizure, hypoparathyroidism	0.42	1.2	1.66	17	3.3	Primary	Calcium supplementation
Orbeck et al. [7]	3 months/F	Irritation, edema, vomiting	0.20	1.4	1.88	16	3.9	Primary	MCT diet, IV albumin/electrolytes
Ozeki et al. [8]	12 years/M	Diarrhea, abdominal pain, edema, weakness, tetany	0.38	–	2.03	14	–	Primary	Low-fat diet, IV albumin, propranolol, everolimus
Troskot et al. [9]	42 years/M	Seizure, edema, diarrhea, weight loss	0.46	1.26	1.78	14	–	Primary	MCT diet, IV albumin/electrolytes, octreotide
Licinio et al. [10]	17 years/F	Edema, ascites, liver fibrosis	↓	–	–	↓	–	Primary	Nutritional therapy
Klingenberg et al. [11	27 years/F	Dyspnea, edema, diarrhea tetany	0.35	1.07	1.49	19	2.82	Primary	Dietary management, IV albumin, octreotide
Gumà et al. [12]	34 years/F	Edema, tetany, extensive warts	↓	↓	–	↓	–	Primary	MCT diet, mineral supplements
Lu et al. [13]	4 years 8 months/F	Diarrhea, edema, tetany	0.92	2.00	2.28	25.8	–	Primary	MCT diet, IV multiple vitamins
Koçak et al. [14]	47 years/F	Malaise, weakness, edema	0.6	1.75	2.38	21	–	Primary	MCT diet
Hennekam et al. [15]	Young man	Lymphedema, facial anomalies, mental retardation	0.65	1.9	2.28	21	3.2	Primary (as a component of Hennekam syndrome)	MCT diet, IV albumin, nutrient supplement
Hennekam et al. [15]	Young women	Edema, seizure, recurrent infections	0.68	1.98	2.44	17	–	Primary (as a component of Hennekam syndrome)	–
Köstel-Bal et al. [16]	2 months/M	Diarrhea, fever, edema	0.46	1.26	1.78	14	–	Primary	MCT diet
Van Biervliet et al. [17]	5 months/M	tonic-clonic seizures, irritability	0.43	–	1.45	25	–	Primary	MCT diet, IV albumin, nutrient supplement
Huppke et al. [18]	10 years/M	Diarrhea, swelling, mental retardation, facial anomaly, muscle weakness	Normal	Normal		Normal		Primary (as a manifestation of Hennekam syndrome)	–
	8 years/M	Mental retardation, facial anomaly, hyperactivity	Normal	Normal		Normal		Primary (as a manifestation of Hennekam syndrome)	–
Hamada et al. [19]	4 months/M	Diarrhea, muscle weakness, convulsion, slow development	0.29	1.15	1.63	16	3.2	Primary	MCT diet, nutrient supplements
Zimmet et al. [20]	33 years/F	Recurrent infections, diarrhea, edema, tetany, lassitude	0.25	1.50	2.18	6	–	Primary	Gluten-free diet, calcium/vitamin D/magnesium supplements
Eisner et al. [21]	13 years/M	Edema, diarrhea, mental retardation, absence of IgA	1.00	2.58	3.05	16.3	–	Primary	Low-fat diet, IV albumin
Scully et al. [22]	70 years/F	Dyspnea, edema, plural effusions	1.6	2.4	2.86	17	2.9	Unlikely primary	–
Bereket et al. [23]	8.5 years/F	Steatorrhea, tetany	0.70	1.73	–	Normal	–	Primary (as a probable component of APS-1)	Steroid replacement, intensive vitamin D supplement, magnesium injection

*IL* intestinal lymphangiectasia, *M* male, *F* female, *MCT* medium chain triglyceride, *IV* intravenous, *APS-1* autoimmune polyglandular disease type 1

*Calculated by total calcium (mmol/L) + 0.02×[40-albumin (g/L)] if not reported

The mechanism by which hypomagnesemia occurs in IL could be manifold. Diarrhea, which occurs in almost every IL case with hypomagnesemia, could result in inadequate magnesium absorption. Since one-third of serum magnesium is bound to albumin, hypoalbuminemia may also disrupt magnesium transportation and balance [24]. Multiple linear regression analyses of the data from Table 2 showed that there were no correlations between serum magnesium levels and total calcium, corrected calcium, or albumin levels (*P* = 0.2620, *P* = 0.6311, *P* = 0.8885, respectively; GraphPad Prism 8, San Diego, USA). Albumin levels do not affect serum magnesium levels. However, due to the small sample size, this conclusion should be interpreted with caution. The Tibetan patient’s diet lacked green leafy vegetables, which are a good source of magnesium, and might have also contributed to the hypomagnesemia in our case.

There could be several potential clinical consequences of hypomagnesemia in IL patients. Severely lowered serum magnesium can cause arrhythmias, including PR interval prolongation, progressive QRS widening, and, most notably, torsades de pointes. Additionally, hypomagnesemia can give rise to neuromuscular hyperactivity, causing tetany and seizures. Hypomagnesemic tetany was reported in one IL patient [20]. Hypokalemia and hypocalcemia are common findings in IL patients. Since magnesium plays a regulatory role in potassium and calcium metabolism, hypomagnesemia could have further complicated these two electrolyte disturbances. In one case, hypocalcemia was recalcitrant to vitamin D and calcium supplementation and was only corrected by magnesium injections [23].

The treatment for hypomagnesemia in IL patients should be individualized. For severe hypomagnesemia with symptoms, intravenous magnesium sulfate is the preferred option. Days may be needed for the restoration of normal concentrations because as much as 50% of the injected magnesium would be lost from the urine. For moderate to mild asymptomatic situations, oral replacement might be adequate [3]. The serum level should be closely monitored, especially in patients with impaired renal function, for whom the risk of hypermagnesemia is high.

In conclusion, hypomagnesemia may be an overlooked complication of IL, and the monitoring of serum magnesium concentration is essential, especially in patients with concomitant neuromuscular and ionic abnormalities, as well as a magnesium-deficient dietary habit.

## Data Availability

All relevant data can be found in the text of this article and the references.

## Abbreviations

IL: Intestinal lymphangiectasia
MCT: Medium chain triglyceride
g: Gram
L: Liter
cm: Centimeter
BMI: Body mass index
kg: Kilogram
m: Meter
ECG: Electrocardiogram
hsCRP: High-sensitivity C-reactive protein
ESR: Erythrocyte sedimentation rate

## References

[1] Huber R, Semmler G, Mayr A, Offner F, Datz C (2020). Primary intestinal lymphangiectasia in an adult patient: a case report and review of literature. World J Gastroenterol.

[2] Alshikho MJ, Talas JM, Noureldine SI, Zazou S, Addas A, Kurabi H (2016). Intestinal lymphangiectasia: insights on management and literature review. Am J Case Rep.

[3] Ahmed F, Mohammed A (2019). Magnesium: the forgotten electrolyte—a review on hypomagnesemia. Med Sci.

[4] Altın Z, Atabay Y, Özer S, Karakoyun M, Ekmekçi S, Yürekli EY (2018). Primary intestinal lymphangiectasia and a review of the current literature. Turk J Gastroenterol Off J Turk Soc Gastroenterol.

[5] Lu J, Zhai H (2017). Exacerbation of primary intestinal lymphangiectasia during late pregnancy and recovery after delivery: a case report and literature review. Medicine.

[6] O’Donnell D, Myers AM (1990). Intestinal lymphangiectasia with protein losing enteropathy, toxic copper accumulation and hypoparathyroidism. Aust N Z J Med.

[7] Orbeck H, Larsen TE, Hovig T (1978). Transient intestinal lymphangiectasia. Acta Paediatr Scand.

[8] Ozeki M, Hori T, Kanda K, Kawamoto N, Ibuka T, Miyazaki T (2016). Everolimus for primary intestinal lymphangiectasia with protein-losing enteropathy. Pediatrics.

[9] Troskot R, Jurčić D, Bilić A, Gomerčić Palčić M, Težak S, Brajković I (2015). How to treat an extensive form of primary intestinal lymphangiectasia?. World J Gastroenterol.

[10] Licinio R, Principi M, Ierardi E, Leo AD (2014). Liver fibrosis in primary intestinal lymphangiectasia: an undervalued topic. World J Hepatol.

[11] Klingenberg RD, Homann N, Ludwig D (2003). Type I intestinal lymphangiectasia treated successfully with slow-release octreotide. Dig Dis Sci.

[12] Gumà J, Rubió J, Masip C, Alvaro T, Borràs JL (1998). Aggressive bowel lymphoma in a patient with intestinal lymphangiectasia and widespread viral warts. Ann Oncol Off J Eur Soc Med Oncol.

[13] Lu Y-Y, Wu J-F, Ni Y-H, Peng SS-F, Chia-Tung S, Chang M-H (2009). Hypocalcemia and tetany caused by vitamin D deficiency in a child with intestinal lymphangiectasia. J Formos Med Assoc.

[14] Koçak G, Koçak E, Akbal E, Duranay M, Köklü S (2011). A rare cause of severe hypoalbuminemia in a patient with primary hypoparathyroidism: intestinal lymphangiectasia. Acta Clin Belg.

[15] Hennekam RC, Geerdink RA, Hamel BC, Hennekam FA, Kraus P, Rammeloo JA (1989). Autosomal recessive intestinal lymphangiectasia and lymphedema, with facial anomalies and mental retardation. Am J Med Genet.

[16] Köstel-Bal AS, Kaymak S, Haskoloğlu Ş, Kuloğlu Z, Ensari A, Doğu F (2016). A clinical approach to a child with hypoalbuminemia and lymphopenia. J Clin Immunol.

[17] Van Biervliet S, Velde SV, Robberecht E, Van Winckel M (2007). Hypocalcaemic seizures: sign of intestinal disease?. Acta Gastro-Enterol Belg.

[18] Huppke P, Christen HJ, Sattler B, Hanefeld F (2000). Two brothers with Hennekam syndrome and cerebral abnormalities. Clin Dysmorphol.

[19] Hamada A, Kondoh T, Kamei T, Tominaga N, Tsuru A, Matsumoto T (2002). Protein-losing enteropathy complicated with recurrent convulsions and developmental delay in a 4-month-old boy. Pediatr Int Off J Jpn Pediatr Soc.

[20] Zimmet P, Breidahl HD (1968). Intestinal lymphangiectasia with hypomagnesaemic tetany. Australas Ann Med.

[21] Eisner JW, Bralow SP (1968). Intestinal lymphangiectasia with immunoglobulin A deficiency. Am J Dig Dis.

[22] Case records of the Massachusetts General Hospital (1984). Weekly clinicopathological exercises. Case 8-1984. An elderly woman with protein-losing enteropathy and pleural effusions. N Engl J Med.

[23] Bereket A, Lowenheim M, Blethen SL, Kane P, Wilson TA (1995). Intestinal lymphangiectasia in a patient with autoimmune polyglandular disease type I and steatorrhea. J Clin Endocrinol Metab.

[24] Liu M, Yang H, Mao Y (2019). Magnesium and liver disease. Ann Transl Med.

